# Associations Between the Apolipoprotein E ε4 Allele and Reduced Serum Levels of High Density Lipoprotein a Cognitively Normal Aging Han Chinese Population

**DOI:** 10.3389/fendo.2019.00827

**Published:** 2019-12-05

**Authors:** Wei Li, Yong Li, Qi Qiu, Lin Sun, Ling Yue, Xia Li, Shifu Xiao

**Affiliations:** ^1^Department of Geriatric Psychiatry, Shanghai Mental Health Center, Shanghai Jiao Tong University School of Medicine, Shanghai, China; ^2^Alzheimer's Disease and Related Disorders Center, Shanghai Jiao Tong University, Shanghai, China; ^3^Department of Psychiatry, Hubei Provincial Hospital of TCM, Wuhan, China

**Keywords:** *APOE* E4, cognitively normal aging, high density lipoprotein, Chinese, cognition

## Abstract

**Background:** Previous studies have confirmed that *APOE* genotype is associated with lipid metabolism, but related studies are inconsistent. Therefore, we conducted this cross-sectional study to explore the associations between apolipoprotein E (*APOE*) genotypes and serum levels of fasting blood sugar, triglycerides, total cholesterol, high density lipoprotein, and low density lipoprotein in a cognitively normal aging Han Chinese population.

**Methods:** One hundred sixty-nine community elders with normal cognitive function were included in the study. Based on multiplex amplification refractory mutation system polymerase chain reaction (PCR), these subjects were divided into three groups: (1) E2/2 or E2/3 (*APOE* E2); (2) E3/3 (*APOE* E3); and (3) E2/4, E3/4, or E4/4 (*APOE* E4). Correlations of serum levels of fasting blood sugar, triglycerides, total cholesterol, high density lipoprotein, and low density lipoprotein with *APOE* genotypes were assessed.

**Results:** The results of Mann-Whitney analysis showed that the concentration of high density lipoprotein (HDL) in *APOE* E2 and E3 groups was higher than that in E4 groups (*p* < 0.05). Logistic regression analysis also suggested that a lower level of high density lipoprotein was associated with the E4 allele (adjusted odds ratio 0.164, 95% confidence interval 0.031~ 0.876, *P* = 0.034).

**Conclusion:** *APOE* E4 is associated with decreased serum high density lipoprotein concentration in healthy elderly. However, the above conclusions need to be further verified.

## Introduction

Apolipoprotein E (APOE) is a polypeptide of 299 amino acids encoded by a gene on chromosome 19. It has three allele isoforms (*APOE* E2, *APOE* E3, *APOE* E4) differing in 112 and 158 amino acids positions (1). The *APOE* E4 allele, found in 13% of the general population, is considered as the predominant risk factor for late-onset Alzheimer's disease (AD); and *APOE* E3, present in most patients, provides an intermediate level of risk; whereas *APOE* E2, found in approximately 10% of the population, confers protection (2). The mechanistic link between *APOE* gene polymorphism and AD has been the focus of numerous studies (3–5), as APOE is one of the primary apolipoproteins in central nervous system (CNS) lipid metabolism (6), we speculate that *APOE* genotype may affect the pathogenesis of AD by altering lipid homeostasis (7). And increasing evidence also supports that the lipidation status of *APOE* plays an important role, more concretely, impacting Aβ aggregation, deposition, and clearance (8). In addition, *APOE* has been shown to directly bind tau, the other major proteinopathy of AD, *in vitro* (9), while neuronal expression of human *APOE* can result in tau hyperphosphorylation *in vivo* (10). So these data also suggest that *APOE* may directly influence tau pathology and tau-mediated neurodegeneration (10).

APOE exists in low-density lipoprotein (LDL) cholesterol as well as high-density lipoprotein (HDL) cholesterol, and HDL is thought to contribute to lipid transport from peripheral tissues to the liver (a process designated as reverse lipid transport) (11). A previous study (12) showed that *APOE* E4 was associated with elevated plasma HDL levels in normal elderly people, and this phenomenon was also demonstrated in patients with dementia (13). However, there were also studies showing that elevated serum HDL levels was associated with a significantly decreased risk of dementia (14, 15). What's more, other studies (16–18) supported that *APOE* E4 was only correlated with a higher serum of total cholesterol and low-density lipoprotein (LDL), but not HDL. Therefore, the conclusions of these studies were inconsistent. Since the relationship between APOE polymorphism with lipid-apolipoprotein blood profiles varies depending on the prevailing regional environmental parameters and ethnicity (19), and there are few similar studies in China, we recruited 169 elderly people from Chinese communities to explore the relationship between *APOE* genotypes and the levels of serum lipids (fasting blood sugar, triglycerides, total cholesterol, high density lipoprotein, and low density lipoprotein).

## Materials and Methods

### Participants

This cross-sectional study included 169 community elderly (age ranges from 60 to 90 years, with an average age of 69.74 ± 7.485; among them, 72 were males, accounting for 42.6%) with normal cognitive function and the method of sampling has been described in our previous studies (20). The inclusion criteria were as follows: (1) aged 60 or more; (2) normal cognitive function; (3) without major medical abnormalities, including central nervous system diseases and unstable, acute or life-threatening medical illness; (4) was able to cooperate and complete relevant inspections. Subjects with a history of major medical abnormalities (e.g., infection and cancer) and mental problems (e.g., schizophrenia, anxiety, depression, mild cognitive impairment (MCI) and dementia) or that might affect cognitive function as well as lipid metabolism were excluded. Through face-to-face interviews, we obtained general demographic data (for example, age and gender), daily living habits (smoking, drinking, drinking tea), and disease history (hypertension and diabetes) of the subjects.

### Clinical Assessment and Cognitive Assessment

In order to exclude depression, mild cognitive impairment, dementia and other mental diseases, all the participants underwent a screening process that included physical and neurological examinations (by an experienced psychiatrist), a review of their medical history, laboratory tests and MRI scans. The Mini-Mental State Examination (MMSE) was used to assess the cognitive function of subjects. Due to the high educational level of most subjects, individuals with MMSE scores >25 were selected as subjects (21). At the same time, we also used the Global Deterioration Scale (GDS) (22) to eliminate depression.

### Genotyping of *APOE* and Biochemical Detection of Blood Lipids

Genomic DNA was extracted from peripheral blood (Morning fasting whole blood) by using a Blood Genomic DNA Extraction Kit (Qiagen NV, Venlo, the Netherlands). And *APOE* genotype was determined by multiplex amplification refractory mutation system polymerase chain reaction (PCR). According to the methods previously described (23), these 169 subjects were divided into three groups, *APOE* E2 (ε2/ε2 and ε2/ε3, *n* = 25), *APOE* E3 (ε3/ε3, *n* = 111), and *APOE* E4 (ε2/ε4, ε3/ε4, and ε4/ε4, *n* = 33), and Supplementary Tables 1, 2 list the information about the gene distribution in detail. By using hexokinase method on an auto-analyzer (Dimension Xpand plus), we obtained the values of serum fasting blood glucose, triglyceride, cholesterol, high density lipoprotein, and low density lipoprotein.

The Research Ethical Committee of the affiliated mental health center of Shanghai jiaotong university school of medicine approved this study, and written informed consent was obtained from all participants before the study. All research processes was conducted in accordance with the principles of Declaration of Helsinki.

### Statistical Analysis

Categorical variables were expressed as frequencies (%) and continuous variables were expressed as mean ± SD or Median. Single sample Kolmogorov-Smirnov test was used to test whether the data conforms to the normal distribution. One-way analysis of variance (ANOVA) Least—Significant Difference (LSD) was used to compare the data of normal distribution among the *APOE* E2 group, *APOE* E 3 group, and *APOE* E 4 group, while Kruskal-wallis H was used to compare the data of non-normal distribution among these three groups. Then Binary regression analysis was used to screen for possible related factors (At this stage, we divided the study population into *APOE* E4 group and non-*APOE* E4 group. And the related variables such as sex, age, and body mass index (BMI) were controlled). Two-tailed tests were used at a significance level of *p* < 0.05 for all analyses. The data were analyzed using SPSS 22.0 (IBM Corporation, Armonk, NY, USA).

## Results

### Characteristic of Subjects With Different *APOE* Genotypes

**Figure 1:**
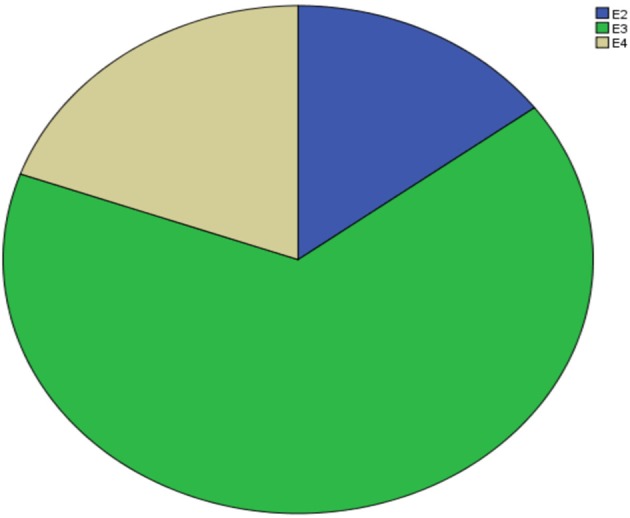
Prevalence of APOE.

**Figure 2:**
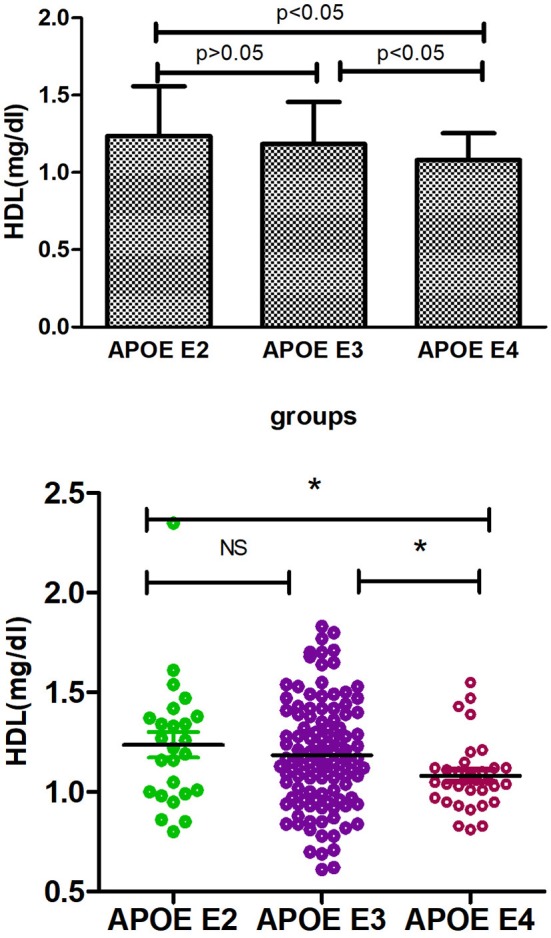
Comparison of HDL among three groups. *p < 0.05; ns, *p* > 0.05.

Supplementary Table 1 shows the results of allele and genotype frequencies. The frequencies of *APOE* E3 were greatest (65.7%), and that of *APOE* E2 and *APOE* E4 was 14.8 and 19.5%, respectively (Figure 1). Supplementary Table 2 shows the characteristic of subjects with different *APOE* genotypes. By using single sample Kolmogorov-Smirnov test, we found that BMI, cholesterol and low density lipoprotein (*p* > 0.05) were in normal distribution, while age, fasting blood sugar, triglyceride, and high density lipoprotein (*p* < 0.05) were in non-normal distribution. By using One-way analysis of variance (ANOVA) Least—Significant Difference (LSD) (Normal distribution) and Kruskal-wallis H (Non normal distribution), we found that there was statistical difference (*p* = 0.001) in high density lipoprotein among the three groups, while there was no significant difference (*p* > 0.05) in age, gender, BMI, hypertension, diabetes, smoker, drinkers, tea drinker, fasting blood sugar, triglyceride, cholesterol, and low density lipoprotein among the three groups. High density lipoprotein in *APOE* E4 group was significantly lower than that in *APOE* E2 group and *APOE* E3 group, while there was no statistical difference between *APOE* E2 group and *APOE* E3 group, Supplementary Table 3 and Figure 2 shows the results (by using Mann-Whitney *U* test). Then we divided 169 elderly people into *APOE* E4 group and non-*APOE* E4 group, by using binary regression analysis and controlling age, gender and BMI, we found that *APOE* E4 was significantly correlated with high density lipoprotein (OR = 0.164, *p* = 0.034. 95%CI 0.031~0.876) (Supplementary Table 4).

## Discussion

*APOE* E4 is the major genetic risk factor for Alzheimer's disease (AD), increasing risk and decreasing age of disease onset (24), and a lot of research (25–27) has demonstrated the detrimental effects of *APOE* E4 in varying cellular contexts. *APOE* is closely related to circulating lipoproteins, specifically high-density lipoproteins, and very low-density lipoproteins (28), and plays an important role in catabolism and transport of lipoproteins (29). Cognitively normal individuals with *APOE* E4 have also been demonstrated alterations in cerebral fatty acid (FA) and carbohydrate metabolism congruent with AD patients (30, 31). Since both *APOE* E4 carriers and individuals with AD exhibit a state of cerebral lipid dyshomeostasis, we hypothesized that APOE may play a role in regulating lipid droplets (LD) metabolism (32).

In this cross-section study, we first investigated the distribution of *APOE* gene polymorphism in Chinese healthy aging adults, and found that the *APOE* E3 genotype and E3 allele were the most prevalent in the elderly people with normal cognition, which was consistent with other studies (18, 33). These differences of APOE allele frequency in the normal elderly population could indicate different disease risks; for example, the E4 allele that confers a higher risk of AD (34). Then we explored the effects of *APOE* polymorphisms on lipid metabolism (including fasting blood sugar, cholesterol, triglyceride, high density lipoprotein, and low density lipoprotein). By using Kruskal-wallis H test and Mann-Whitney *U* test, we found that the concentration of HDL in the serum of *APOE* E4 carriers was significantly lower than that of E2 and E3 carriers (*p* < 0.05), while there was no significant difference (*p* > 0.05) between E2 and E3 carriers. Similarly, there was also no significant difference (*p* > 0.05) in fasting blood sugar, triglyceride, cholesterol, and low density lipoprotein among the three groups. Finally, we further explored the correlation between *APOE* E4 genotype and HDL, and in this step, we combined E2 and E3 into non-E4 groups. By using binary logistic regression analysis, we found that *APOE* E4 was significantly related to HDL and independent of age, gender and BMI (adjusted odds ratio 0.164, 95% confidence interval 0.031~0.876, *P* = 0.034). So we concluded that *APOE* E4 was associated with the decrease of HDL in the serum of normal cognitive elderly.

A previous study has showed that AD patients at the late stage had significantly lower levels of high-density lipids than AD patients at the middle stage, which suggested that the lipid profile might be associated with the development of AD (35). Another research also suggested that low high density lipoprotein was considered as risk factors of dementia in elderly men (36). However, a similar study, also conducted in Chinese cognitively normal aging subjects, found that *APOE* E4 status was significantly correlated with a higher serum level of total cholesterol, but not with high density lipoprotein (18). Therefore, our results are not entirely consistent, and the discrepancy may be due to age differences (the age of our group was significantly lower than that of their group). In addition, genetic and environmental factors may also aggravate the differences (37, 38).

Based on these findings, we can explain why a lower serum level of high density lipoprotein will increase the risk of dementia. First, observational epidemiology studies have found that cardiovascular diseases are associated with AD risk (39), as high-density lipids is a protective factor against coronary atherosclerosis (40), so decreased high-density lipids concentration will increases the risk of coronary atherosclerosis. Second, lower levels of high density lipoprotein can increase the risk of stroke, atherosclerosis and an inflammatory state, which are all related to dementia (41). Third, macular degeneration in the elderly has been associated with impaired cognitive function, AD, and dementia (42). Zeaxanthin is absorbed together with intestinal fat and transported to the retina by high-density lipoproteins, which can as antioxidants, limiting oxidative damage to the retina cells, exerting a protective effect against Age-related macular degeneration (43). Therefore, *APOE* E4 might increase the risk of AD in the elderly by lowering serum levels of high density lipoprotein.

There are also containing some limitations: Our study is a cross-sectional study that fails to establish a causal link between *APOE* E4 and high density lipoprotein. Moreover, relatively small sample size reduces the reliability of research.

## Conclusion

In conclusion, *APOE* E4 is associated with decreased serum high density lipoprotein concentration in Chinese healthy elderly. And further research is required to determine whether these links still exist in longitudinal studies.

## Data Availability Statement

The datasets for this manuscript are not publicly available at the time of publication. Requests to access the datasets should be directed to SX, xiaoshifu@msn.com.

## Ethics Statement

The studies involving human participants were reviewed and approved by The Research Ethical Committee of the affiliated mental health center of Shanghai jiaotong university school of medicine. The patients/participants provided their written informed consent to participate in this study. Written informed consent was obtained from the individual(s) for the publication of any potentially identifiable images or data included in this article.

## Author Contributions

WL, QQ, and LS contributed to the study concept and design. LY and YL acquired the data. XL and SX analyzed the data and drafted the manuscript. All authors read and approved the final manuscript.

### Conflict of Interest

The authors declare that the research was conducted in the absence of any commercial or financial relationships that could be construed as a potential conflict of interest.
