# Supplementary material for: Associations Between the Apolipoprotein E ε4 Allele and Reduced Serum Levels of High Density Lipoprotein a Cognitively Normal Aging Han Chinese Population
Source: Front Endocrinol (Lausanne). 2019 Dec 5;10:827. doi: 10.3389/fendo.2019.00827 (PMC6906139; doi:10.3389/fendo.2019.00827)
Supplement: Supplementary file 1 [file Table_1.DOCX]

Table 1. Allele frequencies and prevalence of APOE Chinese normal cognitive elderly

| APOE | Male(n=72) | Female(n=97) | Combined(n=169) |
| --- | --- | --- | --- |
| E2 (e2/e3) | 11(15.3%) | 14(14.4%) | 25(14.8%) |
| E3(e3/e3) | 45(62.5%) | 66(68.0%) | 111(65.7%) |
| E4(e2/e4,e3/e4, e4/e4) | 16(22.2%) | 17(17.5%) | 33(19.5%) |
| e2/e3 | 11(15.3%) | 14(14.4) | 25(14.8%) |
| e2/e4 | 2(2.8%) | 0 | 2(1.2%) |
| e3/e3 | 45(62.5%) | 66(68.0%) | 111(65,7%) |
| e3/e4 | 13(18.1%) | 16(16.5%) | 29(17.2%) |
| e4/e4 | 1(1.4%) | 1(1%) | 2(1.2) |
