# Supplementary material for: Associations Between the Apolipoprotein E ε4 Allele and Reduced Serum Levels of High Density Lipoprotein a Cognitively Normal Aging Han Chinese Population
Source: Front Endocrinol (Lausanne). 2019 Dec 5;10:827. doi: 10.3389/fendo.2019.00827 (PMC6906139; doi:10.3389/fendo.2019.00827)
Supplement: Supplementary file 2 [file Table_2.DOCX]

Table 2. Characteristics of subjects with different APOE groups

| Characteristics | APOE E2  (n=25) | APOE E3  (n=111) | APOE E4  (n=33) | F | p |
| --- | --- | --- | --- | --- | --- |
| Age,y | 70.40±8.067 | 70.00±7.666 | 68.36±6.388 | 0.720 | 0.488 |
| BMI, kg/M^2^ | 24.22±3.037 | 24.11±3.383 | 24.11±3.281 | 0.013 | 0.987 |
| Fasting blood sugar, mmol/L | 5.146±1.137 | 5.672±1.815 | 5.278±1.249 | 1.404 | 0.249 |
| Triglyceride, mmol/L | 1.903±0.946 | 1.933±1.480 | 2.037±2.012 | 0.071 | 0.932 |
| Cholesterol, mmol/L | 4.935±1.099 | 4.882±1.083 | 4.985±0.958 | 0.129 | 0.897 |
| High density lipoprotein, mmol/L | 1.236±0.321 | 1.184±0.272 | 1.081±0.174 | 2.828 | 0.062 |
| Low density lipoprotein, mmol/L | 2.747±0.839 | 2.906±0.860 | 3.088±0.760 | 1.205 | 0.302 |
| Male,n (%) | 11(44.0) | 45(40.5) | 16(48.5) | 0.335 | 0.716 |
| Hypertension, n(%) | 14(56.0) | 56(50.5) | 17(51.5) | 0.124 | 0.884 |
| Diabetes, n (%) | 2(8.0) | 15(13.5) | 3(9.1) | 0.440 | 0.645 |
| Smoker,n (%) | 6(24.0) | 30(27.0) | 7(21.2) | 0.239 | 0.788 |
| Drinkers, n(%) | 6(24.0) | 24(21.6) | 5(15.2) | 0.416 | 0.611 |
| Tea drinker, n (%) | 11(44.0) | 52(46.8) | 13(39.4) | 0.287 | 0.751 |

Note: Three groups were divided according to APOE genotypes: e2/2 or e2/3 (APOE e2); e3/3 (APOE e3); and e2/4, e3/4, or e4/4 (APOE e4).

Abbreviations: BMI, body mass index;
