# Supplementary material for: Associations Between the Apolipoprotein E ε4 Allele and Reduced Serum Levels of High Density Lipoprotein a Cognitively Normal Aging Han Chinese Population
Source: Front Endocrinol (Lausanne). 2019 Dec 5;10:827. doi: 10.3389/fendo.2019.00827 (PMC6906139; doi:10.3389/fendo.2019.00827)
Supplement: Supplementary file 3 [file Table_3.DOCX]

| Variables | Group 1 | Group 2 | mean deviation | Standard error | p | 95% CI |
| --- | --- | --- | --- | --- | --- | --- |
| High density lipoprotein | APOE E2 | APOE E3 | 0.051 | 0.059 | 0.379 | -0.06~0.17 |
|  |  | APOE E4 | 0.155 | 0.070 | 0.028* | 0.02~0.29 |
|  | APOE E3 | APOE E4 | 0.103 | 0.052 | 0.049* | 0.00~0.207 |

Table 3. Multiple comparisons among three groups
