# Supplementary material for: Associations Between the Apolipoprotein E ε4 Allele and Reduced Serum Levels of High Density Lipoprotein a Cognitively Normal Aging Han Chinese Population
Source: Front Endocrinol (Lausanne). 2019 Dec 5;10:827. doi: 10.3389/fendo.2019.00827 (PMC6906139; doi:10.3389/fendo.2019.00827)
Supplement: Supplementary file 4 [file Table_4.docx]

Table 4. Adjusted effects of APOE e4 allele on high density lipoprotein

| variable | B | S.E. | Wald | df | p | OR | 95% CI |
| --- | --- | --- | --- | --- | --- | --- | --- |
| age | -0.033 | 0.028 | 1.379 | 1 | 0.240 | 0.967 | 0.915~1.022 |
| Male | 0.177 | 0.408 | 0.188 | 1 | 0.664 | 1.193 | 0.537~2.653 |
| BMI | -0.007 | 0.065 | 0.012 | 1 | 0.913 | 0.993 | 0.875~1.127 |
| high density lipoprotein | -1.811 | 0.856 | 4.474 | 1 | 0.034 | 0.164 | 0.031~ 0.876 |

Note: logistic regression model was adjusted for age, gender, body mass index; Abbreviations: OR, odds ratio; CI, confidence interval.
